# PARP-1 dependent recruitment of the amyotrophic lateral sclerosis-associated protein FUS/TLS to sites of oxidative DNA damage

**DOI:** 10.1093/nar/gkt835

**Published:** 2013-09-18

**Authors:** Stuart L. Rulten, Amy Rotheray, Ryan L. Green, Gabrielle J. Grundy, Duncan A. Q. Moore, Fernando Gómez-Herreros, Majid Hafezparast, Keith W Caldecott

**Affiliations:** ^1^Genome Damage and Stability Centre, School of Life Sciences, University of Sussex, Falmer, Brighton, BN1 9RQ, UK and ^2^School of Life Sciences, University of Sussex, Falmer, Brighton, BN1 9QG

## Abstract

Amyotrophic lateral sclerosis (ALS) is associated with progressive degeneration of motor neurons. Several of the genes associated with this disease encode proteins involved in RNA processing, including *fused-in-sarcoma*/*translocated-in-sarcoma* (FUS/TLS). FUS is a member of the heterogeneous nuclear ribonucleoprotein (hnRNP) family of proteins that bind thousands of pre-mRNAs and can regulate their splicing. Here, we have examined the possibility that FUS is also a component of the cellular response to DNA damage. We show that both GFP-tagged and endogenous FUS re-localize to sites of oxidative DNA damage induced by UVA laser, and that FUS recruitment is greatly reduced or ablated by an inhibitor of poly (ADP-ribose) polymerase activity. Consistent with this, we show that recombinant FUS binds directly to poly (ADP-ribose) *in vitro*, and that both GFP-tagged and endogenous FUS fail to accumulate at sites of UVA laser induced damage in cells lacking poly (ADP-ribose) polymerase-1. Finally, we show that GFP-FUS^R521G^, harbouring a mutation that is associated with ALS, exhibits reduced ability to accumulate at sites of UVA laser-induced DNA damage. Together, these data suggest that FUS is a component of the cellular response to DNA damage, and that defects in this response may contribute to ALS.

## INTRODUCTION

Amyotrophic lateral sclerosis (ALS) is caused by progressive degeneration of motor neurons in the spinal cord, brain stem, and motor cortex. Whereas 90% of cases are sporadic (sALS), 10% are familial (fALS) and result from inherited dominant mutations in one of ∼13 genes (1–3). A pervading hypothesis is that most cases of ALS are proteinopathies in which the mutant proteins aggregate in neurons and are toxic. While this can explain the dominant nature of ALS mutations, it does not rule out the possibility that some such mutations act in a dominant negative manner. For example, the mutated proteins in ALS may aggregate and sequester normal proteins, or may inactivate protein complexes in which they are involved, reducing and/or disrupting critical cellular processes necessary for neural function and maintenance. Consistent with this idea, it has emerged recently that several of the genes associated with ALS encode RNA binding proteins involved in mRNA splicing, polyadenylation and stability (4,5). Dominant mutations in one such gene, *fused-in-sarcoma/translocated-in-sarcoma* (FUS/TLS), account for ∼5% of fALS and ∼1% of sALS and are associated with ALS6 (2,6–8). FUS is physically associated with a number of transcription factors and RNA processing proteins, including RNA Polymerase II (RNAP II) (9–14). Depletion of FUS leads to altered expression of numerous genes, and particularly genes with large introns (>100 kb) (4,15). FUS depletion also generates elevated levels of RNAP II harbouring a Ser2-phosphorylated C-terminal domain at transcription start sites (10). Normally this posttranslational modification is associated with elongating RNAP II and is enriched at the 3′-termini of genes.

The above observations are consistent with altered RNA processing being an underlying factor in ALS6. However, if this is the case, it seems likely that the role of FUS extends beyond regulating RNA processing under basal conditions. For example, while FUS binds to ∼70% of gene transcripts, it affects the basal expression level of only a small percentage of these (4,15). One possibility is that FUS might be particularly important to maintain the expression of genes in which transcription is perturbed by cellular stress, such as DNA damage, to prevent inappropriate mRNA processing. DNA suffers from attack by a variety of endogenous sources, including endogenous reactive oxygen species and abortive topoisomerase activity, and as a result accrues DNA lesions that can disrupt transcription. Because the cumulative number of DNA lesions encountered by a gene is dependent on cell age, DNA damage poses a significant threat to gene transcription in long-lived cells such as postmitotic neurons. Consequently, we have examined whether FUS is a component of the response of mammalian cells to DNA damage. We show that FUS is recruited to chromosomal sites of oxidative DNA damage, and that this recruitment is reduced by a mutation that is associated with ALS. Our data highlight a new aspect of FUS function, and support a model in which FUS is recruited to sites of DNA damage to protect or maintain ongoing transcription.

## MATERIALS AND METHODS

### Plasmids and vectors

The mouse Fus (mFUS) cDNA was kindly provided by Abraham Acevedo-Arozena and Peter Joyce (MRC Harwell) and subcloned into peGFP-C3 using HindIII/BamHI. A clone containing the human FUS (hFUS) open reading frame was obtained from Source Bioscience and this was subcloned into peGFP-C3 using polymerase chain reaction (PCR) primers tacgtcgactATGgcctcaaacgatt and cttggatccttTTAatacggcctctc (SalI and BamHI sites underlined, start and stop codons in upper case). The hFUS ORF was also subcloned into pET16b by PCR using PCR primers ccagcatATGgcctcaaacgattat and ttggatccttTTAatacggcctctc (*Nde*I and *Bam*HI sites underlined, start and stop codons in upper case).

### Cells and siRNA

Human A549 and U2OS cells were grown at 37°C in Dulbecco's modified Eagle's medium supplemented with 15% foetal calf serum (FCS), penicillin/streptomycin and l-glutamine (Invitrogen). *PARP-1^+/+^* and *PARP-1^−^^/^^−^* spontaneously immortalized mouse embryonic fibroblasts (MEFs; 16,17) were grown in MEM +10% FCS. siRNA treatment was conducted on human A549 cells using siGENOME smartpool hFUS siRNA (Thermo Scientific M-009497) or a scrambled siRNA control and Metafectene Pro (Biontex) according to the manufacturer’s instructions. Knockdown was achieved by two successive rounds of transfection 24 h apart with 100 pmol siRNA per 5 × 10^5^ cells. Cells were analysed for knockdown 48 h after the second transfection by western blotting using polyclonal rabbit anti-FUS [ProteinTech 11570 at 1:1000 in phosphate buffered saline (PBS) + 1% milk].

### UVA irradiation and immunofluorescence

For direct detection of GFP-FUS, cells were seeded onto glass-bottom dishes (MatTek) 2 days before transient transfection with the indicated expression constructs using Genejuice, as previously described (18). Twenty-four hours after transfection, cells were preincubated for 1 h where shown with 500 nM poly (ADP-ribose) polymerase (PARP) inhibitor KU58948 (kindly provided by AstraZeneca) or 10 µM ATM inhibitor KU55933 (Tocris). Cells were then presensitized with 10 μg/ml Hoechst dye 33258 (Sigma) at 37°C for 30 min. GFP-positive cells were then irradiated with a 351-nm UVA laser focused through a 40×/1.2-W objective using a Zeiss Axiovert equipped with LSM 520 Meta. Ultraviolet UVA (351nm, 0.44 J/m^2^) was introduced to an area of ∼12 × 0.1 μm and images were then captured at 15 s intervals.

For indirect immunofluorescence of endogenous proteins, cells were seeded onto glass-bottom dishes (MatTek) 2 days before microirradiation. Presensitization was carried out as above, and individual cells were irradiated with 4.4 J/m^2^ UVA. Cells were then washed and fixed in PBS + 4% paraformaldehyde, permeabilized in PBS + 0.2% Triton, blocked in 5% bovine serum albumin (BSA) and labelled overnight with rabbit anti-FUS (Proteintech, 1:500 or Sigma, 1:500) and mouse anti-γH2Ax (Milipore, 1:1000) in PBS + 1% BSA. Detection was carried out using Alexafluor anti-mouse 555 and anti-rabbit 488 (Invitrogen; both 1:500 in PBS + 1% BSA) for 1 h before washing and mounting.

### Recombinant proteins

pET16b-FUS was transformed into BL21(DE3)(pLysS) and grown in 500 ml cultures in Luria Broth supplemented with 50 µg/ml ampicillin and 30 µg/ml chloramphenicol to an OD_600_ of 0.6. Expression was induced with 1 mM isopropyl β-d-1-thiogalactopyranoside and cultured for 16 h at 16°C. Soluble protein was extracted by lysis in 20 mM Tris–HCl, pH 7.5, 0.5 M NaCl, 10 mM imidazole, 1% Triton X-100, 2 mM β-mercaptoethanol, then sonicated and clarified by centrifugation. Ni-NTA agarose (Qiagen) was used to purify His-FUS using a 50 mM imidazole wash step and elution with 250 mM imidazole. The full-length protein was further purified by Superdex 200 (30/10) using running buffer (20 mM Tris–HCl, pH 7.5, 0.3 M NaCl, 10% glycerol, 1 mM dithiothreitol). Aprataxin- and PNK-like factor (APLF) was similarly purified.

### Slot blotting

Dilutions of His-APLF and His-FUS were made in PBS and applied to nitrocellulose membrane by vacuum. The membrane was blocked in 5% non-fat milk in binding buffer (BB; 20 mM Tris–HCl, pH 7.5, 100 mM NaCl) for 1 h. 50 nM poly (ADP-ribose) (Trevigen) was incubated with the membrane for 45 min in the above, then rinsed three times with BB for 5 min. The membrane was then incubated with anti-PAR antibody (10 H; Enzo Life Sciences) in BB containing 5% non-fat milk for 2 h and then washed again. Poly (ADP-ribose) (PAR) interactions were detected using anti-mouse horseradish peroxidase-conjugate followed by chemo-luminescence.

## RESULTS

**Figure 1.**
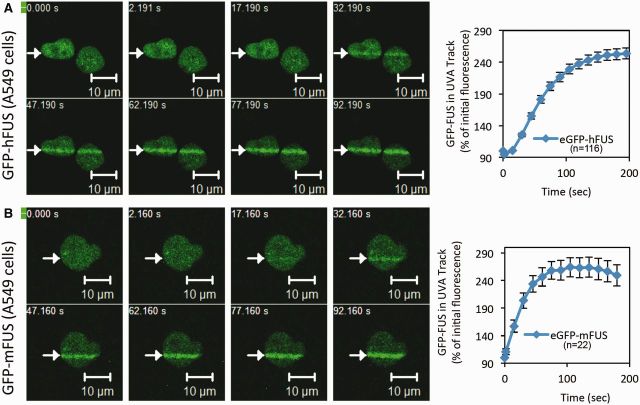
GFP-tagged FUS/TLS accumulates at sites of UVA laser-induced oxidative chromosomal damage. (**A**) Recruitment of eGFP-hFUS to sites of laser damage. Transiently transfected A549 cells were subjected to UVA laser microirradiation along the line indicated. Images were taken at the times (seconds) shown after microirradiation. The graph shows the average GFP fluorescence across six individual experiments and over 100 cells ± SEM. (**B**) Recruitment of eGFP-mFUS to sites of laser damage. Experiments were carried as described in (A). Graph shows the mean of three independent experiments.

**Figure 2.**
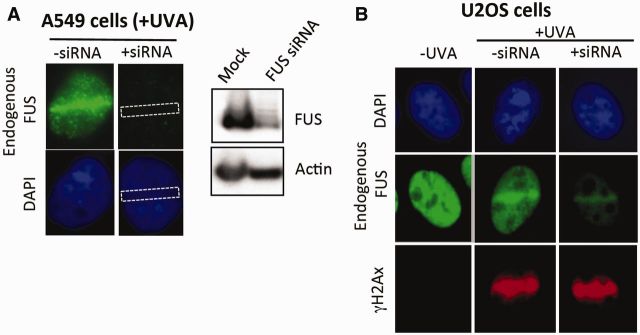
FUS/TLS accumulates at sites of UVA laser-induced oxidative chromosomal damage. (**A**) A549 cells mock-treated (‘−siRNA’) or pretreated with FUS siRNA (‘+siRNA’) were microirradiated, fixed 2 min later and immunostained for endogenous FUS with anti-FUS antibody (top two panels). A western blot confirming siRNA-mediated knockdown is shown (right). The dotted box in the ‘+siRNA’ samples denotes the position of the UVA laser track. (**B**) U2OS cells were treated as described above and immunostained with anti-FUS (middle row), or anti-γH2Ax antibody (bottom row) as a marker of DNA breaks.

To examine whether FUS/TLS is a component of the DNA damage response, we transiently expressed GFP-tagged derivatives of the human and mouse protein in human A549 cells and monitored their subcellular localization before and after DNA damage. Both GFP-hFUS and GFP-mFUS accumulated at sites of oxidative DNA damage induced by UVA laser in A549 cells (Figure 1A and B). This was a rapid response, with GFP-FUS accumulating maximally at sites of DNA damage within 2 min after laser microirradiation (Figure 1A and B, *right panels*). To confirm this was not an artefact of overexpressing GFP-tagged protein, we used anti-FUS antibodies to monitor the subcellular localization of endogenous FUS. Endogenous FUS similarly accumulated at sites of UVA-induced oxidative DNA damage, both in A549 and U2OS cells (Figure 2A and B). Preincubation of the cells with anti-FUS siRNA confirmed that the protein that accumulated at sites of UVA damage was FUS. Immunostaining with antibody that recognize the phosphorylated histone isoform, γH2AX, confirmed that DNA strand breaks were present at the site of UVA laser irradiation (Figure 2B).

**Figure 3.**
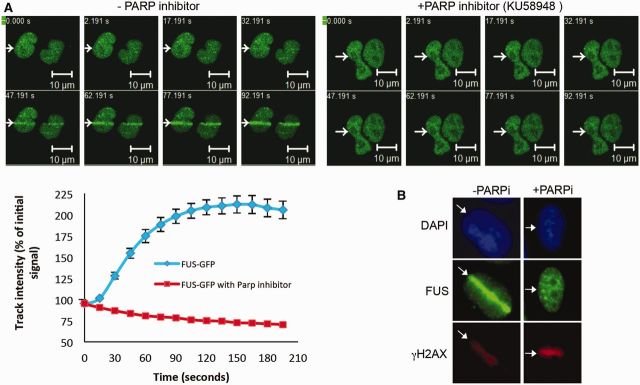
FUS/TLS accumulation at sites of UVA laser-induced damage is dependent on PAR synthesis. (**A**) Human A549 cells were transfected with GFP-hFUS and microirradiated with UVA. Cells were pretreated with vehicle (DMSO) or 500 nM KU58948 1 h before microirradiation. A representative experiment is shown with quantification of GFP-FUS recruitment (mean ± SEM > 30 cells) plotted graphically (bottom left). (**B**) U2OS cells mock-treated or pretreated with 1 µM KU58948 (PARPi) were microirradiated as described above, fixed and immunolabelled for endogenous FUS (middle row) and γH2Ax (bottom).

**Figure 4.**
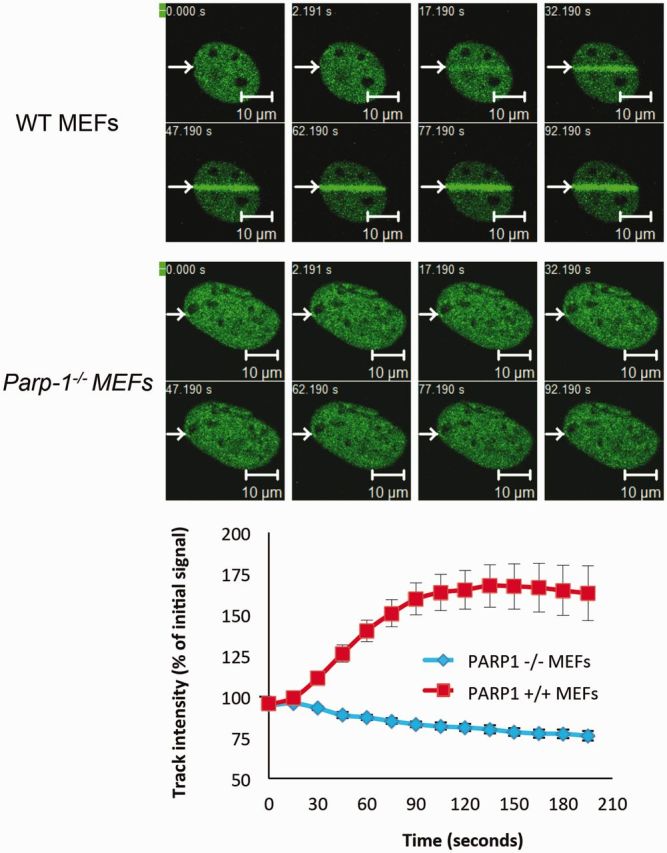
FUS/TLS accumulation at sites of UVA laser-induced damage is dependent on PARP-1. Parp-1^+/+^ (WT) and Parp-1^−/−^MEFs were transiently transfected with GFP-hFUS and microirradiated with a UVA laser. Representative images are presented, with quantification of GFP-FUS recruitment (mean ± SEM > 20 cells) plotted graphically (bottom).

**Figure 5.**
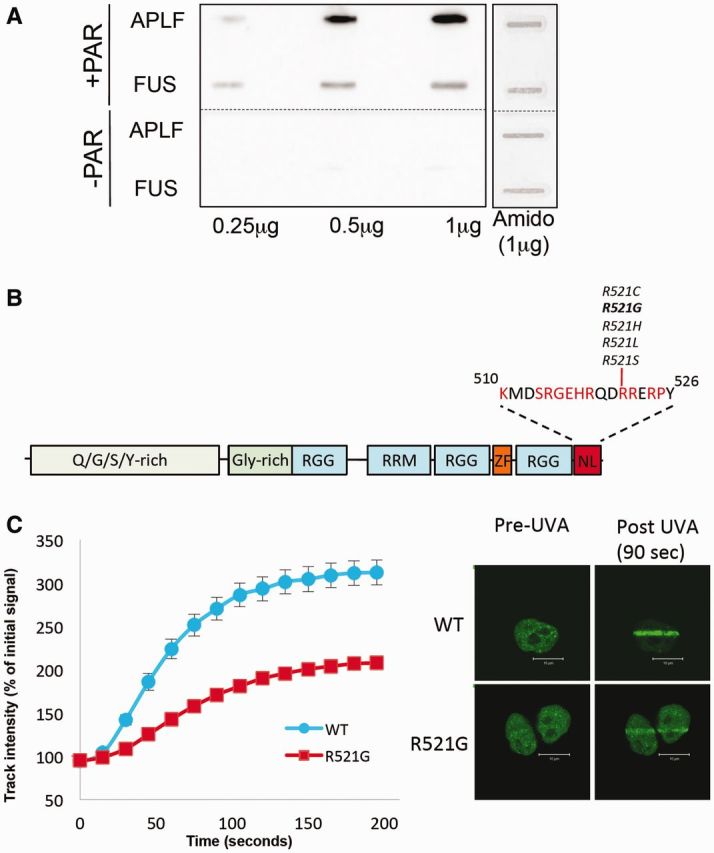
FUS/TLS binds directly to PAR, and a mutation associated with ALS disrupts FUS/TLS recruitment at sites of DNA damage. (**A**) FUS interacts directly with PAR. The indicated amounts of recombinant hFUS or hAPLF were slot blotted onto nitrocellulose membrane and then incubated with (‘+PAR’) or without (−PAR) poly (ADP-ribose). Bound PAR was detected by western blotting. (**B**) Domain structure of FUS/TLS, showing the glutamine/glycine/serine/tyrosine-rich (Q/G/S/Y-rich), glycine-rich (Gly-rich), arginine/glycine-rich (RGG), RNA-binding RRM (RRM), zinc finger (ZF) and nuclear localization (NL) domains. ALS mutations associated with R521 in the NL domain are shown. (**C**) A549 cells transiently transfected with GFP expression construct encoding either wild type (WT) GFP-hFUS or GFP-hFUSR521G were microirradiated with UVA laser and images collected at the indicated times following irradiation. A representative image taken at 90 s following irradiation is shown (right).

A number of proteins that rapidly accumulate at DNA damage sites do so in a manner that is dependent on the synthesis of PAR; a branched nucleic acid-like polymer with which many DNA damage-response proteins bind, resulting in their recruitment and accumulation. We therefore examined whether FUS is similarly recruited at sites of UVA laser-induced oxidative damage in a PAR-dependent manner. Indeed, KU58948, a potent inhibitor of PAR synthesis, prevented the accumulation in A549 cells both of GFP-tagged FUS (Figure 3A) and endogenous FUS (Figure 3B) at sites of UVA laser damage. The source of most (75–90%) cellular of PAR synthesis at sites of DNA damage is poly ADP-ribose polymerase-1 (PARP-1) (18). However, cells possess two additional DNA damage-stimulated PARP enzymes, denoted PARP-2 and PARP-3, both of which are also inhibited by KU58948, albeit to a lesser extent (20–22). To confirm that PARP-1 is required for FUS recruitment, we compared GFP-FUS accumulation at sites of UVA laser damage in wild type and *Parp-1^−^^/^^−^* MEFs. Whereas GFP-FUS rapidly accumulated at sites of UVA laser damage in wild type MEFs, it was unable to do so at such sites in *Parp1^−^^/^^−^* MEFs (Figure 4). Many proteins that are recruited to sites of DNA damage by PARP-1 do so by direct interaction with PAR. To examine if this is the case for FUS, we examined whether recombinant FUS binds to PAR by slot blotting. Indeed, PAR probe bound to slot-blotted recombinant FUS and to recombinant APLF, a known PAR-binding protein and positive control (Figure 5A) (23,24). Together, these experiments suggest that FUS is recruited to sites of oxidative chromosomal DNA damage by the DNA strand break sensor protein, PARP-1, most likely via direct interaction between FUS and PAR.

The C-terminal domain is one of the most commonly mutated regions of FUS, in ALS (1) (Figure 5B). Although this domain influences the nuclear localization of FUS, some ALS-associated mutations in this domain do not appear to impact greatly on FUS nuclear localization (25,26), suggesting that it may fulfill additional functions. We therefore examined whether one of the C-terminal mutations that does not markedly impact on nuclear localization, R521G, might impact on FUS recruitment at sites of transcriptional stress. Indeed, the R521G mutation greatly reduced FUS accumulation at sites of UVA-induced laser damage in A549 cells (Figure 5C). Collectively, these results demonstrate that FUS is a component of the cellular response to DNA damage, and raise the possibility that defects in this response underlie or contribute to ALS.

## DISCUSSION

FUS is a member of the FET family of RNA binding proteins, along with EWSR1 and TAF15, with roles in regulating transcription, splicing and mRNA stability (2,27). FUS binds thousands of pre-mRNA species and regulates the basal level of a small subset of these (4,15,28). In particular, FUS appears to preferentially bind pre-mRNAs with large introns, many of which are expressed in neurons (4,29). The mechanism/s by which dominant mutations in FUS might result in ALS are unclear, however. It has been suggested that FUS mutations, many of which result in sequestration of the mutant protein in the cytoplasm, can form toxic protein aggregates, or that the mutations act in a dominant loss-of-function manner (e.g. by sequestering other proteins into inactive RNA processing complexes) (8,25,30). In both of these scenarios, it is plausible that the outcome relevant to ALS is loss of critical mRNA species. However, because only a few of the target pre-mRNAs bound by FUS exhibit altered basal expression levels in FUS defective cells, we considered the possibility that FUS might play a more important role under conditions of cellular stress, such as following DNA damage.

DNA damage arises continuously in cells, and DNA lesions can disrupt transcription, raising the possibility that RNA processing factors might be differentially regulated in response to DNA damage. Consistent with this idea, a number of RNA processing factors have been reported to accumulate and/or respond to DNA damage, and RNAP II activity, mRNA splicing and polyadenylation are differentially regulated in mammalian cells after DNA damage (31–38). Some RNA processing factors appear to play direct or indirect roles in the repair of DNA strand breaks, by promoting proper expression or recruitment of key DNA repair genes (31,35). In the current study, we observed that GFP-tagged FUS rapidly accumulated at sites of oxidative DNA damage induced by UVA laser, suggesting that this ALS-associated RNA processing factor might also be a component of the cellular DNA damage response.

Exposure to UVA laser induces DNA lesions of the type normally induced by endogenous sources of oxidative stress including DNA base damage and both DNA single- and double-strand breaks. Both mouse and human GFP-FUS accumulated at these sites, confirming that this activity is conserved. Endogenous FUS similarly accumulated at sites of oxidative DNA damage, as measured by two separate anti-FUS antibodies, ruling out that this response was an artefact of overexpressing GFP-tagged protein. We have not detected appearance of GFP-tagged or endogenous FUS in immunofoci following ionizing radiation or H_2_O_2_ (unpublished observations), perhaps indicating that the localization of FUS at sites of DNA damage is transient. Alternatively, it is possible that FUS is only recruited at those sites where transcription is perturbed or blocked, which may be a small subset of DNA damage sites.

A number of proteins that are recruited to sites of DNA breaks do so in a manner that is dependent on synthesis of PAR, the polymeric product of PARP enzymatic activity. The recruitment of GFP-FUS at sites of UVA laser damage was detectable within seconds, and reached a maximum with 2 min, consistent with the rapid kinetics of PAR synthesis. Moreover, FUS accumulation was inhibited by a potent inhibitor of PARP activity and by genetic deletion of PARP-1 in MEFs. Several other RNA binding/processing enzyme are recruited to sites of UVA microirradiation in PARP-1/PAR dependent manner (35,36), suggesting that PARP-1 is a important component of how RNA binding/processing proteins are regulated at sites of oxidative DNA damage.

Many proteins that are recruited to sites of PAR synthesis do so by direct interaction with this polymer. Consistent with this being the case for FUS, recombinant FUS interacted directly with PAR on nitrocellulose slot blots. The RNA processing protein NONO is similarly recruited to sites of UVA microirradiation, via direct interaction between PAR and the RRM1 RNA binding domain (36). The RNA processing proteins ASF/SF2 and a number of hnRNP factors similarly appear to bind PAR directly, via regions encompassing their RRM1 domains (39,40). While this manuscript was in preparation, Mastrocola *et al*. reported result similar to ours, demonstrating FUS recruitment to sites of UVA microirradiation in a PAR dependent manner, although they did not identify the PARP enzyme responsible for this activity (41). However, Mastrocola *et al*. did identify the RGG domains of FUS, rather than the RRM domains, as responsible for PAR binding. It thus appears that several different types of RNA binding domain are able to bind PAR, which while distinct from RNA is related in structure.

Intriguingly, in our hands, a mutation within the C-terminal nuclear localization signal (R521G) greatly reduced FUS accumulation at sites of UVA damage. We do not believe this mutation impacts on PAR binding directly, however, because it is not a component of the RRM or RGG domains, and because we failed to detect such an impact on PAR binding on slot blots, *in vitro* (unpublished observations). Rather, the impact of this mutation on FUS recruitment suggests that other aspects of FUS function are important for its localization at sites of DNA damage, in addition to direct binding to PAR. We do not yet know what this function is, but it is unlikely to reflect an impact on nuclear localization, because the level of nuclear GFP-FUS has been shown to be only weakly affected by this mutation (26), and in our hands was largely unaffected (see Figure 5C). Because this mutation is associated with ALS, however, our data suggest that an inability to localize at nuclear sites of chromosome damage may contribute to this disease in at least some patients.

What role might FUS play at sites of oxidative DNA damage? One possibility is that FUS is recruited to regulate local gene expression. For example, FUS negatively regulates RNA Pol III (42), and is recruited to the *CCND1* promoter following ionizing radiation to inhibit expression of cyclin D1 by promoting localized histone acetylation (43). In the latter case, FUS recruitment is achieved in part by sequence-specific binding of the C-terminus to single-stranded non-coding RNA species transcribed within the vicinity of the *CCND1* promoter. Alternatively, FUS may regulate the stability and/or processing of nascent pre-mRNA at sites of DNA damage. FUS binds to thousands of pre-mRNA species and affects the basal level of a subset of these, most likely by regulating their splicing and/or polyadenylation (4). Alternatively, perhaps FUS is required for efficient repair of oxidative DNA lesions, such as DNA single- or double-strand breaks, as has been described for the RNA processing factors NONO (36), hnRNPUL-1 and -2 (31). Consistent with this notion, FUS is phosphorylated by ATM (44), *FUS^−^^/^^−^* mice exhibit chromosomal instability and radiosensitivity (45,46), and Mastrocola *et al*. reported reduced levels of double strand break repair in FUS depleted cells, using plasmid rejoining/recombination assays (41). However, whether DNA repair defects are present within the context of ALS cells in which FUS mutations are present in a heterozygous and dominant state, remains to be determined.

In summary, we show that FUS is a component of the PARP-1 dependent response to oxidative chromosomal DNA damage, raising the possibility that defects in this response might contribute to ALS.

## FUNDING

Medical Research Council grants [MR/K01854X/1 and MR/J006750/1 to K.W.C.]; We thank Hans and Märit Rausing for their generous support and Scholarship awards (to R.L.G./M.H. and D.A.Q.M./K.W.C.). Funding for open access charge: RCUK block funding.

*Conflict of interest statement*. None declared.
